# Large Friable Rectal Neuroendocrine Tumor Complicated by Hemorrhagic Shock: A Rare Case Report and Review of the Literature

**DOI:** 10.1002/jgh3.70112

**Published:** 2025-02-04

**Authors:** Yazan Abboud, Imran Qureshi, Ayham Khrais, Alexander Le, Param Patel, Ozlem Fidan Ozbilgin, Sima Vossough‐Teehan

**Affiliations:** ^1^ Department of Internal Medicine Rutgers New Jersey Medical School Newark New Jersey USA; ^2^ Division of Gastroenterology and Hepatology Rutgers New Jersey Medical School Newark New Jersey USA; ^3^ Department of Pathology Rutgers New Jersey Medical School Newark New Jersey USA

**Keywords:** endoscopy, NET, rectal neuroendocrine tumor, tumor complication

## Abstract

Rectal neuroendocrine tumors (NETs) are rare neoplasms that are usually asymptomatic and diagnosed incidentally. There are limited data on the presentation and management of high‐grade poorly differentiated large friable rectal NETs. We report the first case of an 87‐year‐old male who presented with chronic diarrhea and subsequently had severe hematochezia complicated by hemorrhagic shock and cardiac arrest and was diagnosed with a 7‐cm high‐grade friable rectal NET. Our case sheds light on the need to consider NETs in cases of chronic diarrhea and on the importance of endoscopy‐guided biopsy in the diagnosis and categorization, which can guide management.

## 1 Introduction

Rectal neuroendocrine tumors (NETs) are rare neoplasms with an incidence rate of 0.17% [1], and median age‐of‐diagnosis of 57 years [2]. Previous data showed that the incidence of rectal NETs has been increasing in the United States, possibly due to the improvement in detection rates in addition to a potential true increase in incidence [3]. Most rectal NETs are asymptomatic and diagnosed incidentally as small yellowish submucosal neoplasms located at the mid‐rectum, at a 4–8 cm distance from the anorectal junction [4]. However, they can induce symptoms and manifest with diarrhea, weight loss, abdominal pain, or gastrointestinal bleeding. The prognosis of rectal NETs varies based on their grade which mainly depends on the mitotic index per 10 high‐power fields and the expression of tumor proliferation marker Ki‐67 [5]. High‐grade rectal NETs are usually poorly differentiated and have aggressive behavior and worse prognosis compared with well‐differentiated tumors. There are limited data on the presentation and management of high‐grade large friable rectal NETs. We report the case of an 87‐year‐old male who presented with chronic diarrhea and subsequently had severe hematochezia complicated by hemorrhagic shock and cardiac arrest and was diagnosed with a 7‐cm high‐grade friable rectal NET which was managed using a multidisciplinary approach including endoscopy, interventional radiology, and chemoradiation therapy.

### 1.1 Case Report

**FIGURE 1:**
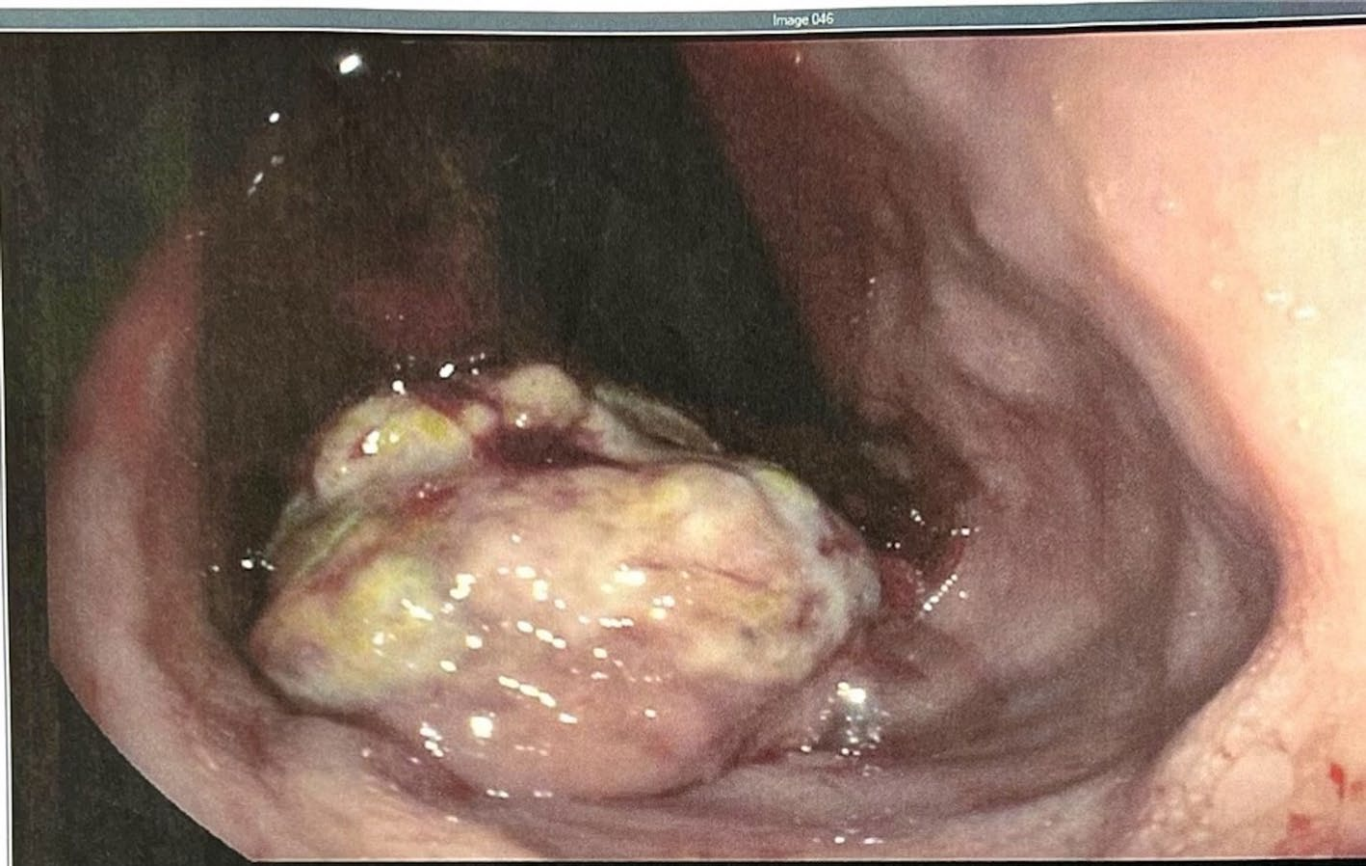
Large 5‐cm friable ulcerative rectal neuroendocrine tumor involving the anal verge and dentate line and extending to the perirectal area.

**FIGURE 2:**
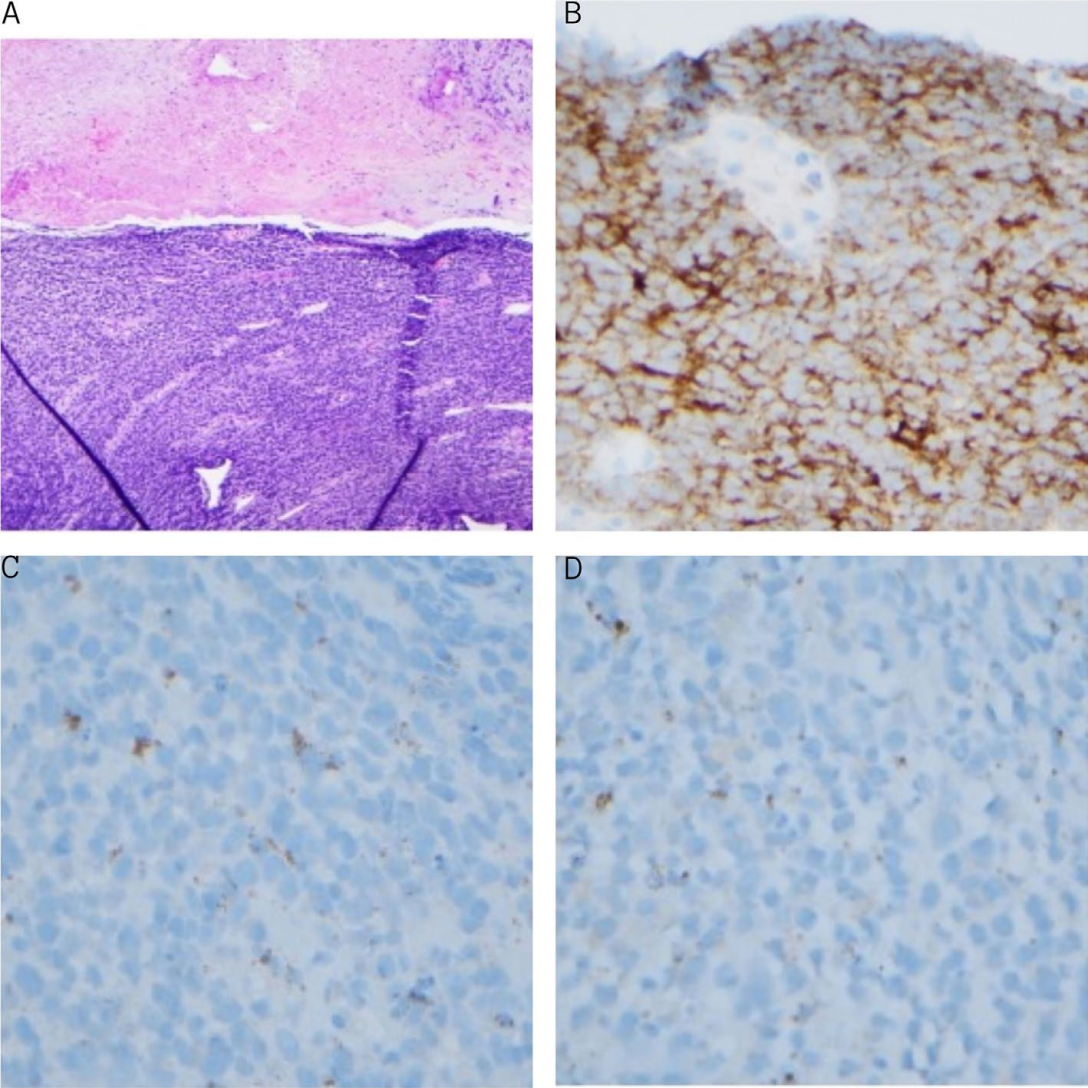
(A) H&E stain demonstrating infiltrative neuroendocrine carcinoma with necrosis on the top. (B) Positive CD‐56 staining. (C) Patchy positive synaptophysin. (D) Negative chromogranin.

An 87‐year‐old male with history of colon cancer and subsequently diverticulosis requiring left hemicolectomy with sigmoid resection, presented with a 5‐month history of diarrhea and fecal incontinence, along with 10‐pound unintentional weight loss over the last month. The diarrhea was mostly watery with episodes of dark brown cottage cheese‐like stool. The patient denied any recent travel, sick contacts, or antibiotic use. Physical exam was unremarkable and lab workup showed normocytic anemia (Hemoglobin of 11 g/dL and MCV of 91 fl). Routine workup for evaluation of chronic diarrhea was non‐diagnostic, yielding the decision to perform a colonoscopy and an esophagogastroduodenoscopy. On presentation, the patient had a CEA level of 6.6 ng/mL which increased to 8.6 ng/mL in a week. The patient also had a high chromogranin‐A level of 630.6 ng/mL. The patient was noted to have bright blood per rectum with clots pouring on the bed in the endoscopy room. While the esophagogastroduodenoscopy did not reveal any bleeding, the colonoscopy showed bleeding located mostly in the distal 30 cm of the colon; 1:10 000‐epinephrine was injected which controlled the bleeding. An ulcerated and friable rectal mass involving the anal verge and dentate line and extending to the perirectal area, which was likely the source of bleeding, was noted and biopsied (Figure 1). During the following night, the patient had > 20 episodes of small‐volume hematochezia, leading to hemorrhagic shock complicated by cardiac arrest; he was resuscitated with massive transfusion protocol. Computed tomography (CT) of the abdomen and pelvis showed evidence of focal high attenuation focus in the rectum, suggestive of active bleeding, with enlarged peri‐rectal lymph nodes, and a large presacral mass measuring 6.8 × 4.8 cm. He then underwent embolization of the right internal iliac artery which resolved the hematochezia. Histopathological analysis of the mass was consistent with a neuroendocrine tumor, demonstrating predominantly ulceration and necrosis. Immunohistochemistry staining showed positive CD56, positive p16, patchy positive Synaptophysin, and perinuclear dot staining of Pankeratin (Table 1 and Figure 2). CDX2, CK7, CK20, CD45/LCA, TTF‐1, and Chromogranin staining were negative. The Ki‐67 proliferation index was approximately 80% and there was necrosis as well as increased mitotic activity. This was a poorly differentiated NET, demonstrating small cell neuroendocrine carcinoma morphology, and was classified as WHO grade 3 [6]. The patient subsequently underwent five sessions of inpatient styptic radiation therapy for a total dose of 2500 cGy. The patient was then discharged to a sub‐acute rehabilitation facility and underwent positron emission tomography CT which showed metastasis to the liver and pancreas. He was then started on chemotherapy with carboplatin AUC5 and etoposide 100 mg/m^2^. The patient underwent two cycles of chemotherapy with each cycle repeating after 21 days. During each cycle, the patient received 370 mg of carboplatin on Day 1 and 185 mg of etoposide on Days 1, 2, and 3. Two weeks after his second cycle of chemotherapy, a repeat CT scan was done showing progression of the disease. The patient elected to go for hospice care and passed away a month later.

**TABLE 1:** Immunohistochemistry clones.

CD56	MRQ‐42
Synaptophysin	MRQ‐40
Chromogranin	LK2H10
CDX2	EPR2764Y
TTF‐1	SP141
P16	Cinte
CK7	SP52
CK20	SP33
CD45/LCA	CRP2118
KI‐67	(30–9)
Pankeratin	AE1/AE3/PCK26

## 2 Discussion

Rectal NETs are rare tumors that are usually small and asymptomatic [4]. To the best of our knowledge, this is the first reported case of a large friable rectal NET that was complicated by hemorrhagic shock. Chronic diarrhea and anemia in our patient prompted further investigations using endoscopy procedures yielding the diagnosis of rectal NET during the colonoscopy. Our current case demonstrates the importance of clinical correlation between lower gastrointestinal symptoms and endoscopy‐guided biopsy in the diagnosis of rectal NET. Furthermore, we demonstrate a multidisciplinary approach to managing a rare complication of this tumor.

The clinical presentation of NETs can vary depending on their location in the gastrointestinal tract. For instance, Smith et al. retrospectively reviewed 126 patients with high‐grade NETs and showed that patients with anorectal NETs presented at an earlier stage, compared to patients with colonic NET who presented at a later stage, likely due to the higher likelihood of developing symptoms such as hematochezia in anorectal NETs [7]. While our patient did not present initially with hematochezia, he endorsed diarrhea and was anemic, and subsequently developed hematochezia prior to diagnosing the rectal NET. However, despite being diagnosed earlier, patients in the aforementioned study did not have better outcomes. While 92% of patients had a clinical response to chemotherapy with or without radiation, the disease recurred in all patients. Surgery remains an option for patients who fail to respond to chemoradiation therapy; however, it is unclear if it improves overall prognosis given its risk of morbidity and mortality [7]. Our patient underwent chemoradiation therapy after a multidisciplinary discussion.

Rectal NET management is tumor‐specific and depends on size, grade of differentiation, tumor proliferative index, and lymphatic and vascular invasion. Endoscopic features of rectal NETs such as ulcerations or erosions are rare but when present can indicate a more aggressive disease [8]. These features can be complicated by bleeding which can affect the management approach of the tumor. The rectal NET in the current case was friable and ulcerative, and complicated by hemorrhagic shock, prompting the need for endoscopic management and subsequent embolization via interventional radiology. Guidelines regarding best therapeutic approaches for high grade poorly differentiated localized rectal NETs are not well established with few similar cases reported in the literature (Table 2); however, radiation with platinum‐based chemotherapy, using a combination of etoposide and cisplatin remains the treatment of choice [9], with disparities in treatment outcomes depending on tumor proliferation marker Ki‐67 [10]. We hope that our case adds to the literature on this topic and provides a possible therapeutic approach to such a presentation.

**TABLE 2:** Review of literature of similar cases.

Source	Age (years), sex	Symptoms at presentation	Endoscopic features	Biopsy findings	Treatment	Outcomes
Suyama et al. [11]	57, M	Hematochezia, constipation	Polypoid lesion and a large tumor in the rectal wall about 2 to 3 cm from the anal verge (12.8 × 9.2 cm)	Positive for chromogranin A, synaptophysin, CD56, and somatostatin receptor–3; MIB‐1 index was very high, at approximately 70%, neuroendocrine cells characterized by a proliferation of small‐sized carcinoma cells with hyperchromatic nuclei and scanty cytoplasm with delicate fibrovascular stroma	Total pelvic exenteration, adjuvant chemotherapy using cisplatin and camptothecin‐11	No recurrence 6 months after surgery
Misawa et al. [12]	53, F	Anal pain	Submucosal tumor in the lower rectum	Large multilocular cystic tumor, measuring 8 cm in diameter in the retrorectal space of the pelvis, submucosal tumor in the lower rectum, 8 × 7 × 5 cm in greatest dimension, extended into the adventitia and muscularis propria of the rectum and consisted of 2 components, highly cellular, high‐grade tumor faces the central cystic space, whereas the lower grade tumor is less cellular and located at the periphery	Abdominoperineal resection with bilateral lymph node dissection was performed to remove the tumor, fluid in the tumor cyst was aspirated through the rectal wall yielding 200 mL of a bloody serous discharge	Four months after resection, local recurrence appeared and a total dose of 64‐Gy radiation was delivered, tumor partially responded, but multiple recurrent tumors appeared in the pelvis and lungs at 8 months after resection, died 11 months after the operation
Torre et al. [13]	42, M	Hematochezia	Ulcerated lesion 12 cm distant from the anal verge	Small‐cell poorly differentiated NEC pT3N1 (6/14), G3, Ki67 98%	3 cycles of first‐line chemotherapy with cisplatinum (CDDP 75 mg/m^2^ on Day 1, every 3 week) and etoposide (VP16 100 mg/m^2^ on Days 1–3, every 3 week), sphincter‐sparing open anterior low rectal resection, and fashioning of a colonic terminal stoma	Relapse in a month treated with systemic chemotherapy with FOLFIRI (irinotecan) 180 mg/m^2^ on Day 1, followed by folinic acid 400 mg/m^2^, a 5‐FU bolus of 400 mg/m^2^ and 5‐FU 1200 mg/m^2^ in a 44‐h infusion days (1–2 cycles every 14 days) for 12 cycles followed by pelvic radiotherapy; additional radiotherapy after 8 months for disease in lymph nodes
Miyamoto et al. [14]	56, M	Hematochezia	Type 4 lesion mainly located on the right side of the lower rectum	Poorly differentiated neuroendocrine tumor positive for chromogranin A and synaptophysin; Ki‐67 of 87.8%	Low anterior resection with lymph node dissection and total meso‐rectal excision	Multiple recurrent tumors in pelvis and lung 8 months after the operation; death 1 year after operation

In conclusion, our case sheds light on the importance of endoscopy‐guided biopsy in the diagnosis and categorization of rectal NETs, which can guide management. Clinicians should consider rectal NETs in the differential diagnosis of chronic diarrhea and hematochezia, which should be managed in a timely manner to avoid any subsequent sequelae. Future studies are warranted to further evaluate the best therapeutic approach to poorly‐differentiated large rectal NETs.

## Consent

Informed patient consent was obtained for publication.

## Conflicts of Interest

The authors declare no conflicts of interest.
